# Unexpected Subglottic Stenosis Detected During Difficult Intubation for the Induction of General Anesthesia: A Case Report

**DOI:** 10.7759/cureus.103177

**Published:** 2026-02-07

**Authors:** Shota Aoki, Hiromasa Kida, Kan Takahashi, Fumiya Sawasaki

**Affiliations:** 1 Anesthesiology, Kanazawa Medical University, Ishikawa, JPN; 2 Anesthesiology, Shiga University of Medical Science, Shiga, JPN

**Keywords:** bronchoscopy, difficult airway, laryngoscopy, pediatric anesthesia, subglottic stenosis

## Abstract

Subglottic stenosis presents with a wide spectrum of symptoms depending on the severity of the stenosis, which may remain completely asymptomatic and therefore escape routine preoperative evaluation. We report a seven-month-old girl who experienced unanticipated failed tracheal intubation despite an optimal Cormack-Lehane grade I laryngoscopic view. Multiple cuffless endotracheal tubes of progressively smaller sizes encountered identical resistance immediately below the vocal cords, while oxygenation and ventilation remained adequate. Recognizing the possibility of a fixed subglottic lesion, further intubation attempts were avoided. Airway management was safely achieved using a supraglottic airway device, allowing the procedure to proceed without hypoxemia or airway trauma. Postoperative bronchoscopic examination revealed whitish membranous stenosis in the subglottic area. This case underscores that optimal glottic visualization does not guarantee successful intubation in infants and highlights the importance of early recognition of fixed subglottic resistance, timely transition to supraglottic airway devices to prevent airway injury, and prompt bronchoscopic evaluation in unexpected pediatric difficult airways.

## Introduction

Subglottic stenosis is a pathological narrowing of the subglottic region extending from immediately below the vocal cords to the inferior border of the cricoid cartilage. It is the third most common airway anomaly, following laryngomalacia and vocal cord paralysis [1]. In infants, the subglottic region is anatomically the narrowest portion of the airway; therefore, even minimal narrowing can result in a marked increase in airway resistance. Approximately 5% of subglottic stenosis cases are congenital, and 95% are acquired [2]. Congenital subglottic stenosis is further classified into membranous and cartilaginous types [3]. Clinical manifestations range from asymptomatic cases to stridor and severe respiratory distress requiring tracheostomy immediately after birth [4].

In mild or asymptomatic cases, a congenital laryngeal web is often difficult to diagnose preoperatively through physical examination or imaging studies. It may first present as unexpected difficult intubation during the induction of general anesthesia. Inadequate airway evaluation may result in significant perioperative risks, including difficulty with mask ventilation, failed intubation, postoperative airway edema, and the need for reintubation.

Membranous congenital subglottic stenosis generally presents with mild symptoms, making preoperative diagnosis difficult in asymptomatic patients.

Here, we report a case of membranous congenital subglottic stenosis that was diagnosed postoperatively following unanticipated difficult intubation during induction of general anesthesia.

Although several reports describe difficult intubation caused by congenital laryngeal webs and subglottic stenosis, cases involving completely asymptomatic infants with a Grade I laryngoscopic view yet total failure of tracheal intubation are uncommon. This scenario presents unique challenges for anesthesiologists and highlights the limitations of routine preoperative assessments.

## Case presentation

A 7-month-old female infant (height: 66.7 cm, weight: 7.9 kg) was scheduled to undergo surgery for right thumb polydactyly. She was born at 37 weeks and 1 day of gestation via spontaneous vaginal delivery, with a birth weight of 2.42 kg. No other congenital anomalies or medical history were noted.

**Figure 1:**
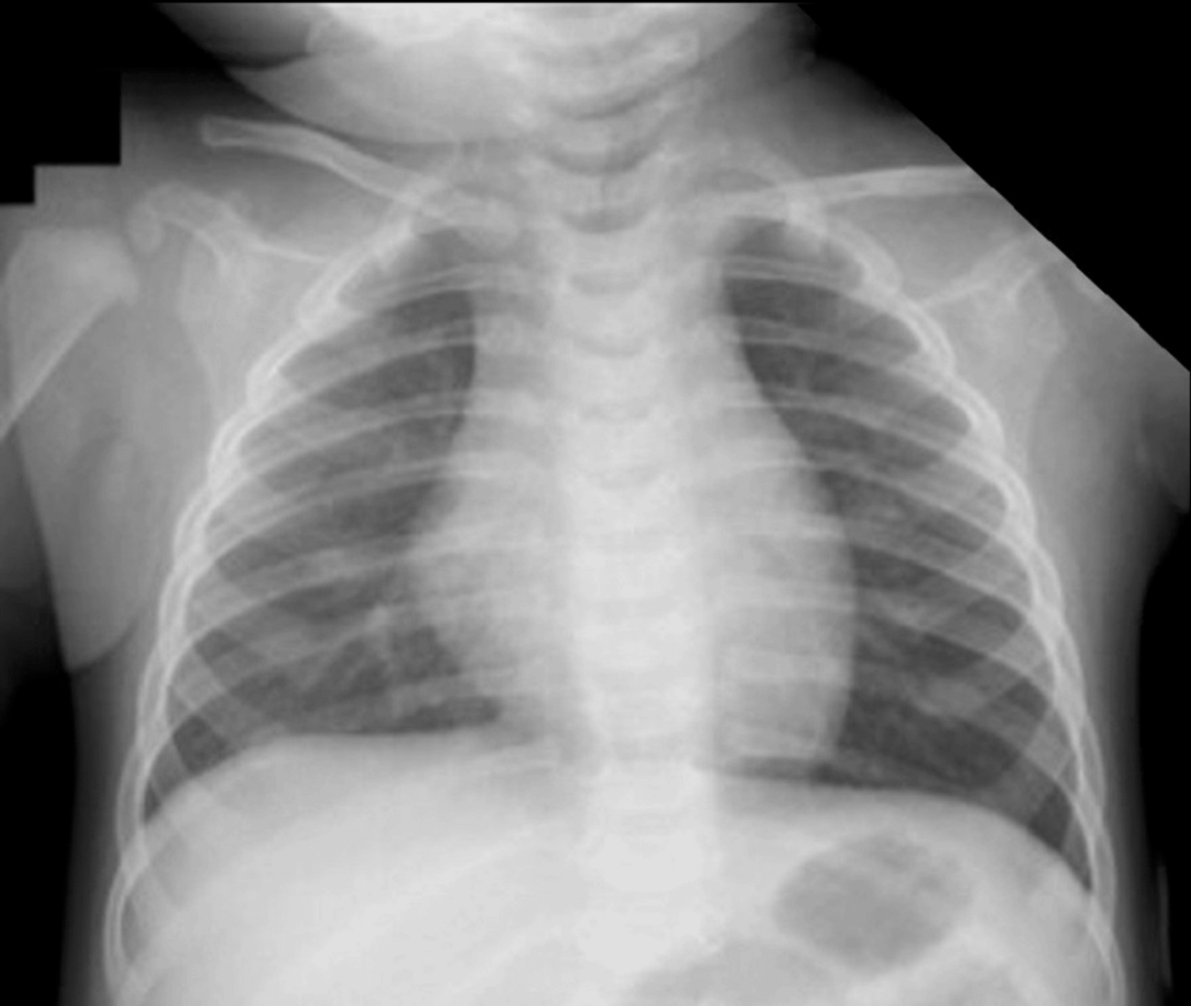
Preoperative chest X-ray The preoperative chest X-ray shows no abnormal findings in the lung field and mediastinum.

Preoperative physical examination revealed a mouth opening of approximately 1.5 fingerbreadths, with no visible abnormalities in the oral cavity. There was no evidence of chest retractions, and breath sounds were clear bilaterally without adventitious sounds. Preoperative chest X-ray revealed no significant abnormalities in either lung field or mediastinum (Figure 1). 

Premedication was administered 30 minutes prior to room entry and consisted of rectal administration of a mixture of midazolam (2.5 mg) and 4 ml of normal saline. No respiratory compromise was observed despite the sedative state.

Anesthesia was induced using oxygen, nitrous oxide, and sevoflurane, followed by the intravenous administration of rocuronium (8 mg) and atropine sulfate (0.08 mg). Mask ventilation was achieved without difficulty. Laryngoscopy with a McGrath® video laryngoscope (Medtronic, Minneapolis, Minnesota, US) provided a Cormack-Lehane Grade I view. A cuffless 3.5 mm internal diameter endotracheal tube was advanced; however, resistance was encountered immediately after the tube tip passed the vocal cords. Despite rotating the tube to adjust the direction, the same resistance was felt, prompting the removal of the tube and resumption of mask ventilation. No anatomical abnormalities or foreign bodies were visible on the McGrath® monitor during laryngoscopy.

Subsequent attempts with three different cuffless tubes (ID: 3.0 mm, 2.5 mm, and 2.0 mm) also encountered resistance just below the vocal cords. A supraglottic airway device (i-gel® size 1.5) was inserted to secure the airway. Given the short duration of surgery, absence of upper airway obstruction symptoms preoperatively, and ease of mask ventilation following induction, we concluded that the airway could be safely maintained with a supraglottic device. Moreover, in the event of airway edema-induced stenosis, prompt detection and management would be feasible in the operating room setting. After consultation with the surgical team, the procedure was carried out as planned.

No desaturation was observed during airway management. To mitigate potential airway edema from repeated laryngoscopy and intubation attempts, 50 mg of hydrocortisone was administered after securing the airway. Intraoperative oxygen saturation remained between 99% and 100%, with no increase in airway pressure or hypoventilation. The surgical time was 2 hours, and the total anesthesia time was 4 hours and 7 minutes. The patient emerged smoothly from anesthesia, and the supraglottic airway device was removed. Post-emergence vital signs were stable (BP: 90/72 mmHg, HR: 126 bpm, SpO₂: 99%), with no signs of respiratory distress such as chest retraction or stridor.

**Figure 2:**
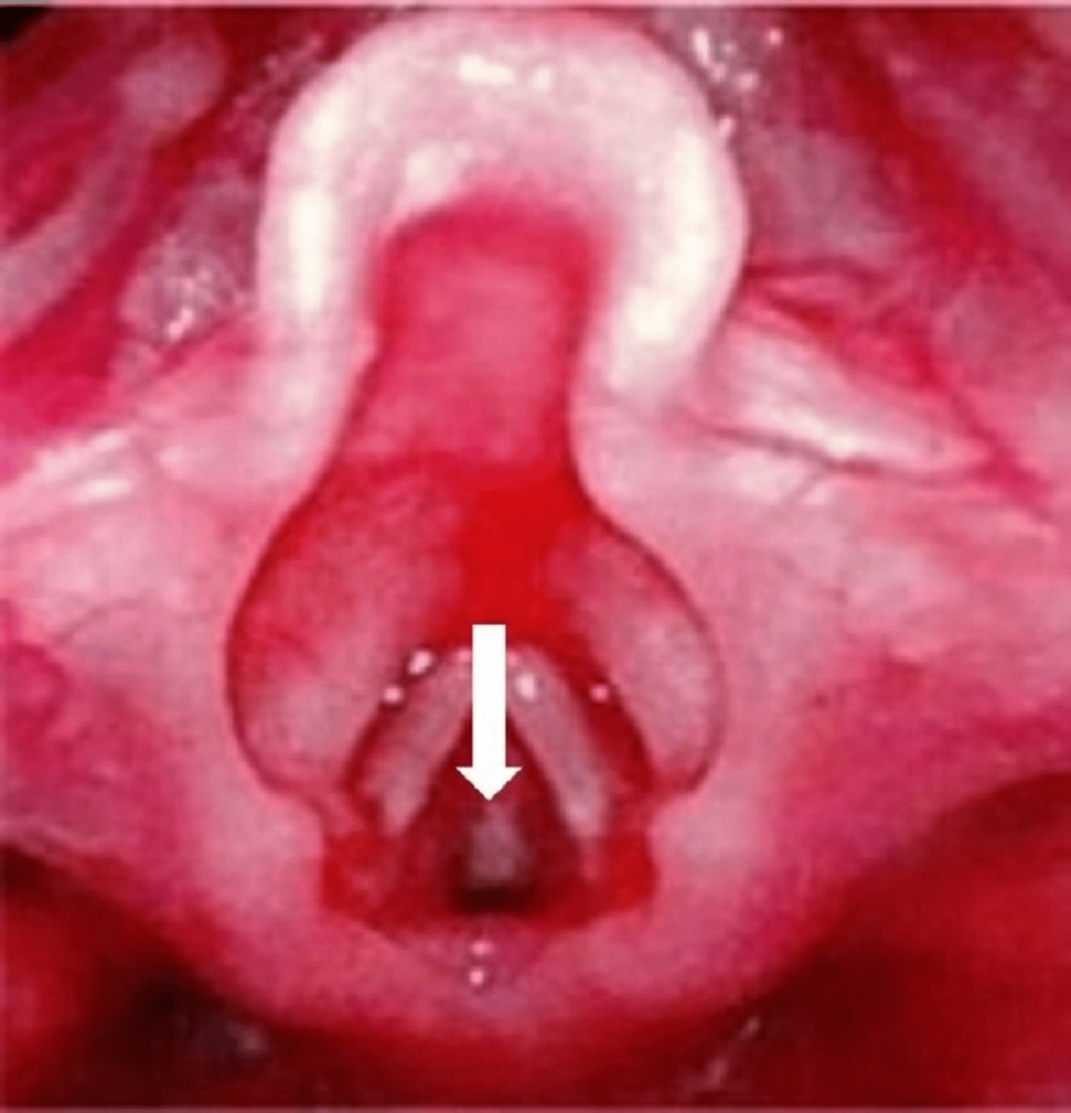
Subglottic findings observed during laryngo-bronchoscopy A whitish membrane was observed in the subglottic region (white arrow).

After returning to the ward, laryngo-bronchoscopy was performed by the otolaryngologists to investigate the cause of the unexpected difficult intubation. A whitish membranous structure was observed just below the vocal cords (Figure 2).

**Figure 3:**
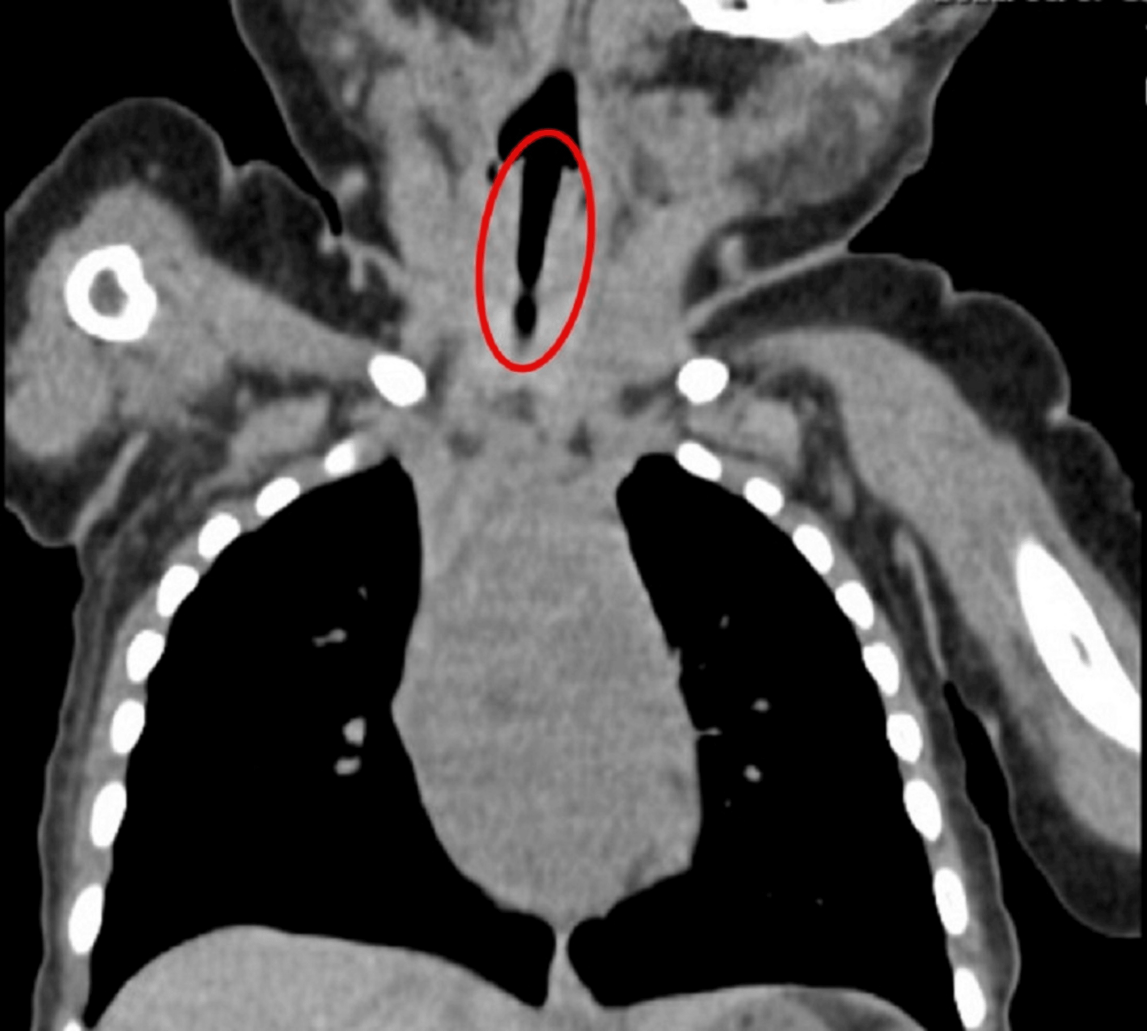
Findings on cervical CT No deformity and stenosis involving tracheal cartilage was observed (red circle).

On postoperative day 1, head and neck CT using 3-mm slice thickness revealed no stenosis or deformity in the identifiable anatomical structures from the larynx to the trachea (Figure 3).

However, the use of 3-mm CT slice thickness may have limited our findings, as thinner slices are recommended for a more detailed assessment of the laryngeal and glottic regions. Nevertheless, the postoperative course was uneventful, and the patient was discharged on postoperative day 3.

## Discussion

Subglottic stenosis is a pathological narrowing of the airway extending from immediately below the vocal cords to the inferior border of the cricoid cartilage and may be congenital or acquired. Approximately 95% of cases are classified as acquired, most commonly resulting from tissue scarring or granuloma formation following airway trauma [5]. Among these traumatic causes, prolonged endotracheal intubation accounts for approximately 90% of cases leading to subglottic stenosis [6].

Congenital subglottic stenosis is further classified into membranous stenosis, caused by thickening of the mucosa and soft tissues, and cartilaginous stenosis, resulting from malformation or hypertrophy of the cricoid cartilage. Membranous subglottic stenosis is generally associated with relatively mild clinical symptoms [3]. Definitive diagnosis requires direct laryngoscopy or bronchoscopic examination [3,7]. The severity of pediatric subglottic stenosis is commonly assessed using the Myer-Cotton classification. In cases of mild stenosis (Grade I-II) without clinical symptoms, conservative management with observation may be selected, anticipating airway enlargement with growth [8]. In the present case, the patient was classified as Grade II and remained asymptomatic.

In undiagnosed, asymptomatic patients, it is difficult to detect subglottic stenosis using routine preoperative evaluations such as physical examination or CT imaging. Consequently, the condition may only be discovered when unanticipated difficult intubation occurs following anesthesia induction. However, when the lesion is membranous in nature and classified as Myer-Cotton Grade I-II, the risk of airway collapse or obstruction during positive-pressure ventilation using a face mask or supraglottic airway device is low. Therefore, airway management using supraglottic devices can generally be performed safely. When abnormal resistance is encountered during tracheal intubation, clinicians should include subglottic stenosis in the differential diagnosis and manage the airway accordingly. In the present case, an earlier bronchoscopy following supraglottic airway placement may have facilitated a prompt diagnosis.

On the other hand, definitive surgical correction should be prioritized for severe symptoms. Surgical management depends on the severity of the stenosis, with endoscopic balloon dilation (EBD) recommended for mild cases [9], whereas laryngotracheoplasty (LTP) is indicated for severe cases [10]. If surgical procedures requiring general anesthesia are necessary before definitive repair, preoperative planning should include the possibility of surgical airway access. In emergencies, clinicians must be vigilant about the potential need for immediate diagnosis and surgical airway management in the event of failed intubation.

Even if tracheal intubation is possible, it may cause post-extubation airway edema, leading to upper airway obstruction, respiratory distress, or hypoxemia. Therefore, selecting an appropriately sized endotracheal tube is essential. Postoperative management in an intensive care setting should be considered if signs of upper airway obstruction develop after extubation.

In the present case, despite attempts with multiple endotracheal tube sizes, tracheal intubation was unsuccessful, and the airway was finally secured with a laryngeal mask. In retrospect, bronchoscopy should have been performed immediately after securing the airway with the supraglottic device. Early endoscopic evaluation would have provided a definitive diagnosis and avoided unnecessary tube exchanges.

## Conclusions

We report a case of subglottic stenosis that was discovered due to difficult intubation during general anesthesia induction and successfully managed using a supraglottic airway device. In asymptomatic cases, preoperative identification is challenging. Therefore, when abnormal resistance is encountered during endotracheal tube placement, subglottic stenosis should be considered in the differential diagnosis. This case provides important educational lessons for anesthesiologists. Recognition of abnormal subglottic resistance should prompt early cessation of repeated intubation attempts and immediate consideration of a supraglottic device to avoid mucosal injury and potential postoperative airway obstruction.
